# Transport of charged small molecules after electropermeabilization — drift and diffusion

**DOI:** 10.1186/s13628-018-0044-2

**Published:** 2018-03-21

**Authors:** Esin B. Sözer, C. Florencia Pocetti, P. Thomas Vernier

**Affiliations:** 1 Frank Reidy Research Center for Bioelectrics, Old Dominion University, 4211 Monarch Way, Ste. 300, Norfolk, VA 23508 USA; 2 Department of Bioengineering, Instituto Tecnológico de Buenos Aires, Buenos Aires, Argentina

**Keywords:** Electropermeabilization, YO-PRO-1, Propidium, Calcein, Small molecule transport, Diffusion, Drift, Electrodiffusion, Membrane transport

## Abstract

**Background:**

Applications of electric-field-induced permeabilization of cells range from cancer therapy to wastewater treatment. A unified understanding of the underlying mechanisms of membrane electropermeabilization, however, has not been achieved. Protocols are empirical, and models are descriptive rather than predictive, which hampers the optimization and expansion of electroporation-based technologies. A common feature of existing models is the assumption that the permeabilized membrane is passive, and that transport through it is entirely diffusive. To demonstrate the necessity to go beyond that assumption, we present here a quantitative analysis of the post-permeabilization transport of three small molecules commonly used in electroporation research — YO-PRO-1, propidium, and calcein — after exposure of cells to minimally perturbing, 6 ns electric pulses.

**Results:**

Influx of YO-PRO-1 from the external medium into the cell exceeds that of propidium, consistent with many published studies. Both are much greater than the influx of calcein. In contrast, the normalized molar *efflux* of calcein from pre-loaded cells into the medium after electropermeabilization is roughly equivalent to the *influx* of YO-PRO-1 and propidium. These relative transport rates are correlated not with molecular size or cross-section, but rather with molecular charge polarity.

**Conclusions:**

This comparison of the kinetics of molecular transport of three small, charged molecules across electropermeabilized cell membranes reveals a component of the mechanism of electroporation that is customarily taken into account only for the time during electric pulse delivery. The large differences between the influx rates of propidium and YO-PRO-1 (cations) and calcein (anion), and between the influx and efflux of calcein, suggest a significant role for the post-pulse transmembrane potential in the migration of ions and charged small molecules across permeabilized cell membranes, which has been largely neglected in models of electroporation.

**Electronic supplementary material:**

The online version of this article (10.1186/s13628-018-0044-2) contains supplementary material, which is available to authorized users.

## Background

Biomedical, industrial, and environmental applications based on electroporation — the electric-field-driven breakdown of the cell membrane barrier function — include electrochemotherapy [1], gene electrotransfer therapy [2], calcium electroporation [3, 4], tumor ablation [5, 6], food processing [7], and waste-water treatment [8]. Optimization and extension of these technologies is hindered by the present lack of knowledge of the biomolecular structure of the electropermeabilized membrane and of the mechanisms of electroporative molecular transport [9]. For plasmid DNA, a multi-stage, multi-component scheme for electrotransfer has been proposed [10], but except for the relatively small amount of material transferred *during* pulse delivery, electroporative transport of inorganic ions and small molecules, which occurs largely across the permeabilized membrane *after* pulse exposure [11–14] is still commonly considered to be essentially passive diffusion through aqueous pores similar to those observed in molecular dynamics simulations [15, 16].

The fluorescent dyes propidium and calcein have been used for decades as implicitly equivalent small-molecule indicators of electroporation [17, 18], even though one is a cation with a fluorescence that is greatly enhanced after polynucleotide binding and the other is an anion, natively fluorescent in aqueous solution. YO-PRO-1, a more sensitive detector of membrane permeabilization by ultra-short (nanosecond) electric pulses (and a cation and a nucleic acid intercalator like propidium), has become the nanoelectroporation indicator of choice [19, 20]. Experimental studies and models of electroporative small molecule transport rarely mention, however, the large quantitative differences between the influx of propidium (and YO-PRO-1) and that of calcein into cells after similar pulsed electric field exposures, and (with one exception [18])) the large difference between calcein *influx* from the medium into a permeabilized cell and calcein *efflux* from a pre-loaded cell that is then permeabilized.

Previous studies reporting quantitative measurements of electroporative transport (quantitative in terms of amounts of material, not fluorescence intensity) have in general looked at only one small-molecule species, typically propidium (or related compounds like ethidium), calcein (or Lucifer yellow, another natively fluorescent anion), or more recently YO-PRO-1, so there has been no direct comparison of the electroporative transport of cationic, nucleic acid-binding dyes with anionic, natively fluorescent dyes. And because of the lack of consistency in pulse exposures, cell types, dye concentrations and loading protocols, and imaging and photometric procedures, a given published quantitative study of propidium transport, for example, can be compared with another study of calcein transport only in relative and very approximate terms. The (mostly overlooked) clue in the literature that there is something very different about the transport of these two classes of compounds is that protocols call for much higher concentrations of calcein (or Lucifer yellow) than for propidium (or YO-PRO-1) [11, 21].

With this narrow methodological focus, it has thus been possible without too much difficulty to interpret individual studies of small molecule electroporative transport in the framework of the “standard model” of electroporation — passive diffusion through electric-field-generated, transient, aqueous pores in the cell membrane [22–24] — with ad hoc adjustments of model parameters. This perpetuates the status quo, in which the various implementations of this “standard model” remain descriptive (they can represent experimental observations after the fact, case by case) instead of becoming predictive (which would greatly enhance the practical value of the models).

A recent modeling study simulated transport of both propidium and calcein, but, in the absence of comparable reports on experimental observations, fell prey to the longstanding treatment of propidium and calcein as implicitly equivalent [25]. That is, in the computations for post-pulse transport, the electrical charge of these molecules (propidium: + 2; calcein: − 4) was assumed to effect only the partitioning between the aqueous medium and the lower permittivity membrane interior, and this in turn was assumed to depend only on the Born energy, determined by the electrostatic energies in the two regions [26]. This analysis neglects two, important, charge-dependent phenomena.

First, in addition to the Born energy, which comes from the dielectric properties of the pore and membrane material, the contribution of the membrane dipole potential (potential in the membrane interior relative to the bulk medium, arising from water and phospholipid dipole orientation) should not be overlooked. Commonly accepted to be on the order of several hundred millivolts positive, the membrane dipole potential increases the transport energy barrier for cations and lowers it for anions [27].

Second, the effect of even a small, non-zero, post-pulse transmembrane potential cannot be ignored, and accumulating experimental evidence indicates that substantial recovery of membrane resting potential occurs within seconds after electropermeabilization [14, 28].

Why has this not received more attention from both experimentalists and modelers?

Modelers can only confront and represent experimental data when it is available to them. The dearth of reports of quantitative comparisons of cationic and anionic small molecule transport (e.g., propidium and calcein) is at least partly responsible for the widely-accepted assumption that the transmembrane potential in electropermeabilized cells can be ignored after the porating pulse has ended, and that therefore there is no drift (electrophoretic) component to post-pulse transport [21, 25, 29–32].

Despite one early warning to the contrary [33], an incorrect assumption — membrane conductivity increases and remains elevated after electroporation, therefore the voltage across the membrane must be negligible — has dominated thinking about post-pulse transport of charged species. This assumption is still commonly held, even when models take account of charge in the interactions of transported molecules with the membrane and pore interface.

The transmembrane potential at any instant is determined by both the conductivity of the membrane *and* the current across the membrane. The cellular response to membrane permeabilization includes, among many other stress- and damage-induced processes, restoration of osmotic balance and physiological K^+^, Na^+^, Ca^2+^, and Cl^−^ concentration gradients. This involves ion pump and channel activation and associated ion currents. If the permeabilized cell has sufficient energy reserves, it is reasonable to expect that it can achieve a new homeostasis that includes a significant non-zero membrane resting potential long before the compromised membrane barrier functions have been repaired and normal conductivity has been established [14, 28]. Rigorous quantitative studies that confirm or refute this hypothesis are needed.

**Fig. 1:**
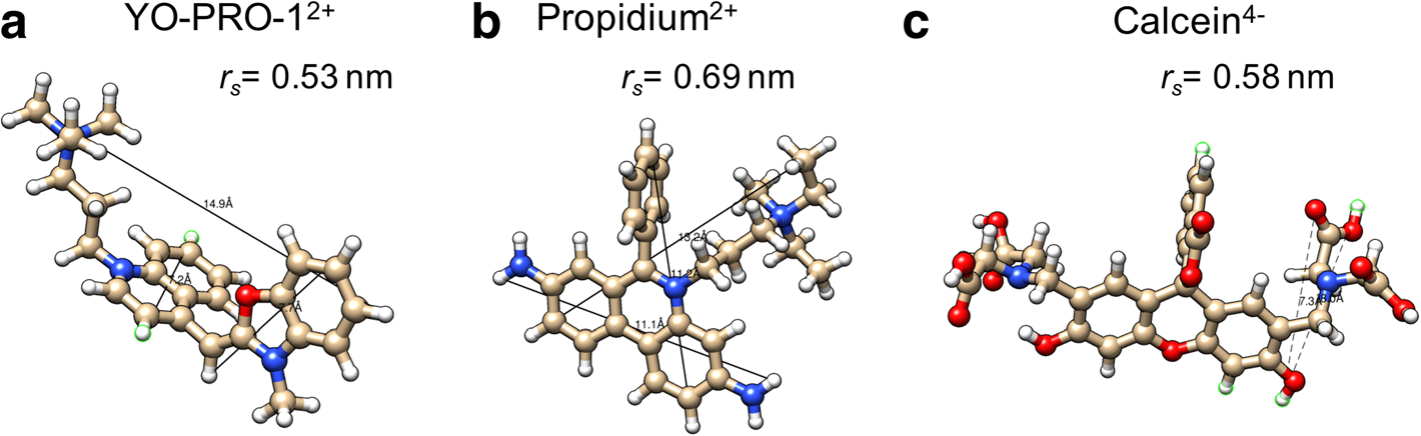
Molecular structures of YO-PRO-1 (**a**), propidium (**b**), and calcein (**c**)

Recently we reported quantitative measurements of YO-PRO-1 transport kinetics following membrane permeabilization by a single 6 ns electric pulse [12]. Here we extend these observations to compare transport of YO-PRO-1, propidium, and calcein, three molecules of similar size, but different charge polarity (Fig. 1). In our imaging-based experiments, detection of YO-PRO-1 and propidium transport into the cell requires that the dye molecule cross the membrane, migrate in the intracellular medium until it contacts a polynucleotide helical region (RNA or DNA), intercalate between base pairs of the polynucleotide helix, and exhibit the resulting fluorescence enhancement. We expected that YO-PRO-1 and propidium influx kinetics would be similar, and we wondered whether calcein influx data, which is not dependent on the transported molecule locating and binding to a target, might be a more direct measure of transmembrane traffic of small molecules after membrane permeabilization.

We provide a direct quantitative comparison of molecular transport of three similarly sized but chemically different fluorescent indicators of membrane permeabilization — two cationic molecules (YO-PRO-1 and propidium), and one anionic molecule (calcein). Our results show that the influx of YO-PRO-1 and propidium (both cations) into electropermeabilized cells is an order of magnitude greater than that of calcein (an anion) after the same pulse exposure. Calcein *efflux* from cells loaded before permeabilization, however, is similar in magnitude to YO-PRO-1 and propidium *influx*. These results are consistent with a significant drift component of post-pulse transport of small charged molecules across electropermeabilized cell membranes, driven by the rapid recovery of transmembrane potential after permeabilization as shown by analytical calculations based on Nernst-Planck electrodiffusion.

## Methods

### Cells

U-937 (human histiocytic lymphoma monocyte; ATCC CRL-1593.2) cells [34] were cultured in RPMI-1640 medium (Corning® glutagro™ 10–104-CV) with 10% fetal bovine serum (Corning, 35–010-CV) and 1% penicillin/streptomycin at 37 °C in a humidified, 5% CO_2_ atmosphere.

### Pulsed electric field exposure

**Fig. 2:**
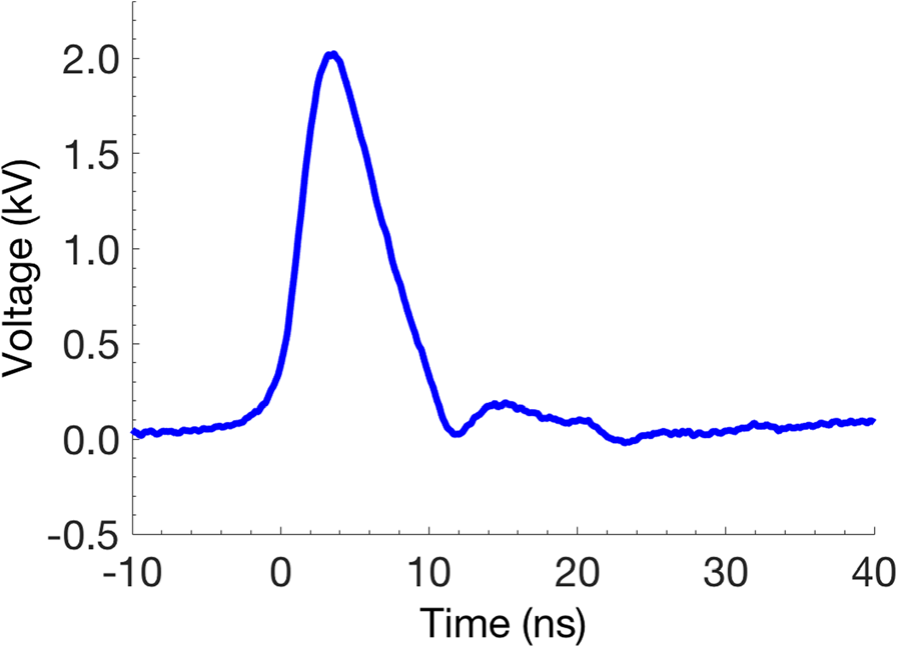
Typical 6 ns waveform recorded during application to a cell suspension

6 ns, 20 MV/m pulses (FID pulse generator FPG 10-10NK) were delivered to cells in suspension in cover glass chambers (Nunc™ Lab-Tek™ II) through parallel tungsten wire electrodes with a 100 μm gap [35]. Cells were observed at laboratory temperature on the stage of a laser scanning confocal microscope (Leica TCS SP8), 8–10 min after they were transferred to the cover glass chambers. A typical pulse waveform can be seen in Fig. 2.

### Imaging

Laser scanning confocal fluorescence microscope images were captured (LeicaTCS SP8) every 100 ms (YO-PRO-1 and propidium) or every 200 ms (calcein) for two minutes (1200/600 frames) from cell suspensions at room temperature in ambient atmosphere on the microscope stage. Emission/excitation peaks are 491/509 nm for YO-PRO-1, 535/617 nm for propidium, and 495/515 nm for calcein. Cells were exposed to electrical pulses five seconds after the start of the recording unless otherwise stated. For z-stack measurements with calcein in Tyrode’s solutions, cells were imaged ±12.5 μm from their approximate central focal plane for 26, 1.5 μm thick z-steps separated by 1 μm. Acquisition of each z-stack takes 70 s; a total of 7 z-stacks are recorded in 530 s.

### Image processing

Cells visible in the microscope field between the electrodes were manually selected for fluorescence photometric image analysis before each pulse exposure. Fluorescence intensities of each region of interest were extracted using custom MATLAB routines that allow tracking of cells in a series of frames. The following built-in MATLAB functions were used in custom image processing routines: ‘imroi’, for manually choosing regions of interest; ‘regionprops’, for evaluating geometric properties of regions of interest.

### Molecular transport imaging

For imaging of fluorescent dye influx, cells were washed and suspended at approximately 5 × 10^5^ cells/mL in fresh medium containing either 2 μM YO-PRO-1, 30 μM propidium, or 200 μM calcein. For calcein efflux imaging, cells were loaded in fresh medium containing 0.5 μM calcein-AM for 15 min at 37 °C in a humidified, 5% CO_2_ atmosphere, then resuspended in fresh RPMI 1640 medium. Fig. 1 shows the molecular structure and size of each dye. For experiments with regular and high-K^+^ Tyrode’s solutions, cells were washed and suspended in either regular Tyrode’s with 140 mM NaCl, 5.4 mM KCl, 10 mM glucose, 2 mM CaCl_2_, 1.5 mM MgCl_2_, or high-K^+^ Tyrode’s where NaCl is replaced with KCl with a final KCl concentration of 145 mM. About 10 min after resuspension, imaging was initiated and pulses were delivered to the cells. At least 30 cells from three independent experiments are analyzed for each reported data set unless otherwise indicated.

### Calibration

**Fig. 3:**
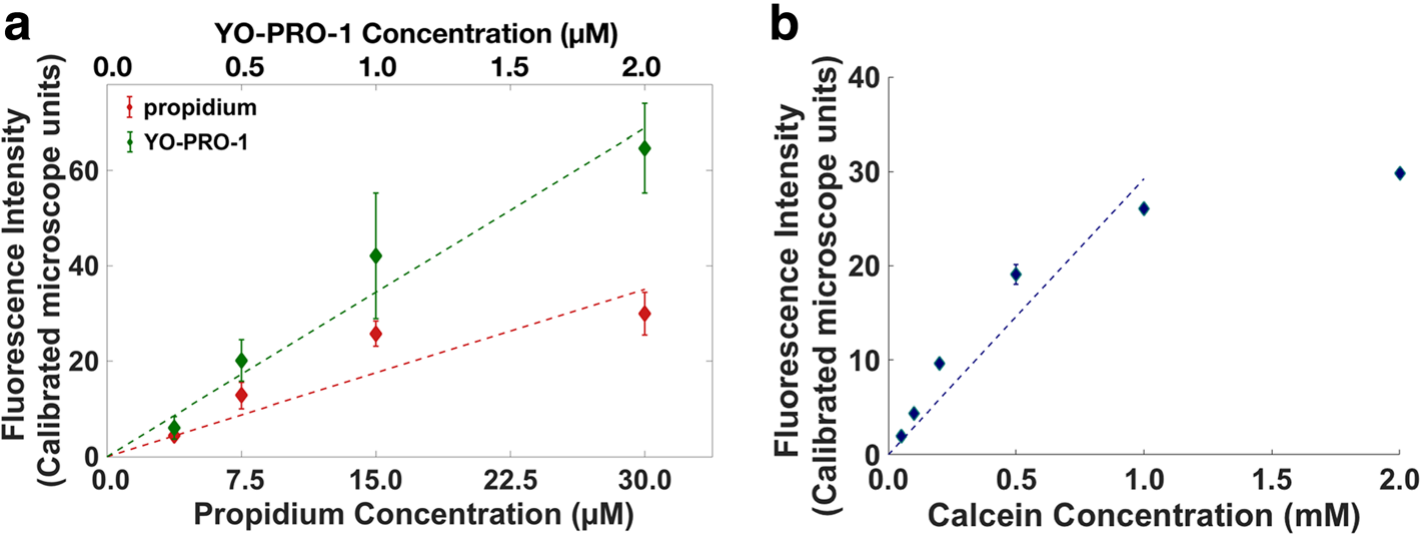
Calibration curves for (**a**) YO-PRO-1 (top x-axis) and propidium (bottom x-axis), and (**b**) calcein. The non-linearity for calcein at higher concentrations is caused by fluorescence quenching. Error bars are standard deviation

The procedure for correlating propidium and YO-PRO-1 fluorescence to molar concentration closely follows a method previously described [12, 20]. Dense lysates were created from U-937 cells (8 × 10^7^ cells/mL) by adding 0.1% Triton X-100 and then sonicating for 2 min with a Misonix Sonicator S-4000, 1 s alternating on-off cycles, amplitude 20. Calibration curves were generated by adding known concentrations of the dye to the lysate (Fig. 3a). Each point on the curves represents measurements taken in triplicate from three separate preparations. For calcein influx measurements, the fluorescence of the extracellular medium (200 μM calcein) in each experiment was measured in three cell-free regions; the mean of these measurements was taken to be the fluorescence intensity of 200 μM calcein. Measurements for other calcein concentrations in cell-free preparations show that calcein fluorescence is linear with concentration up to approximately 1 mM (Fig. 3b). Quenching is observed at higher concentrations. The linear region was used for calibration of concentration in calcein efflux experiments, where cells were loaded with calcein-AM as described above.

### Z-stack measurements

To report as accurately as possible the changes in intracellular concentrations of the three dyes, cell volume changes resulting from nanosecond pulse permeabilization-induced swelling [36–38] and osmoregulatory responses to high K^+^medium must be incorporated into the calculations. We used confocal z-stack-based measurements of cell volume from calcein-loaded cells (where the cell outline is sharply defined by the fluorescence boundaries) for volume normalization in all data sets, since the electropermeabilizing pulse doses were the same for the observations with all three dyes. Here we summarize this procedure.

Cell regions in each z-step were isolated by thresholding the fluorescence images using the built-in Matlab function ‘graythresh’. First, the total cell area for each cell over the z-stack is added to get the initial, cell-volume-proportional sum of pixels (${V}_{c, pixelsum,{t}_0}$). This value is related to the initial cell volume by a proportionality constant *α*.

1 $$ {V}_{c, pixelsum,{t}_0}={\sum}_{\mathrm{zsteps}}{A}_{c,\mathrm{pixel},{t}_0} $$

2 $$ {V}_{c,{t}_0}=\alpha {V}_{c, pixelsum,{t}_0} $$

**Fig. 4:**
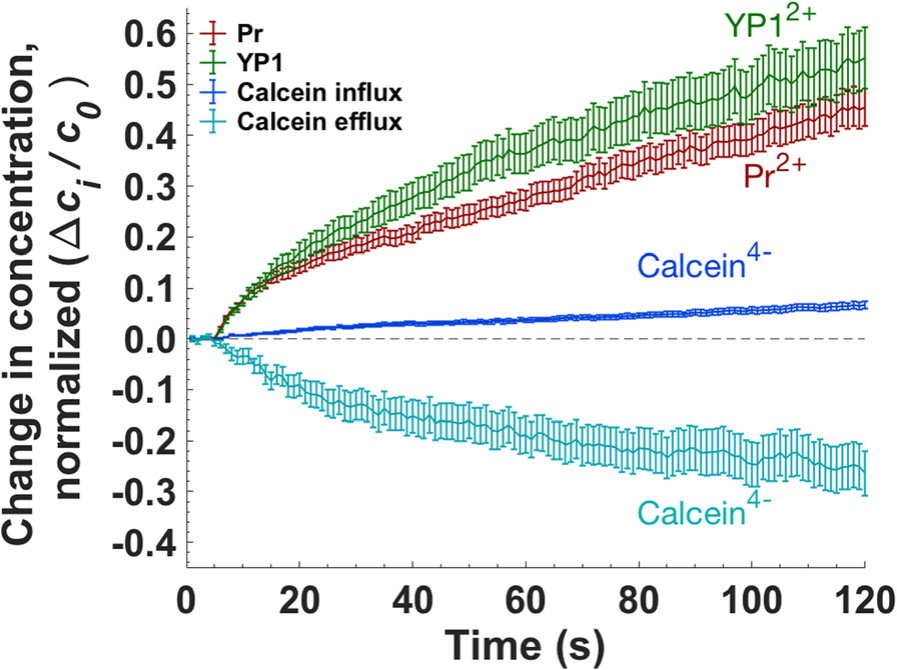
YO-PRO-1 (YP1), propidium (Pr), and calcein influx, and calcein efflux after 10 pulses, 20 MV/m, 1 kHz repetition rate. Pulse delivery begins at *t* = 5 s. *n* ≥ 30, in 3 experiments

The cell volume change *V*_*c*, *t*_ at any time point *t* relative to the volume ${V}_{c,{t}_0}$ at the initial time *t*_*0*_ is then given by ${V}_{c,t}/{V}_{c,{t}_0}={V}_{c, pixelsum,t}/{V}_{c, pixelsum,{t}_0}$. From the calcein z-stacks we extracted the relationship between these *volume* changes and the changes in *area* observed for the mid-cell confocal slices, which under the conditions of these experiments is *V*_2_/*V*_1_ = (*A*_2_/*A*_1_)^1.2^. This relationship was used to estimate volume changes for the cells in the propidium and YO-PRO-1 experiments, where volumes could not be accurately determined from z-stacks, and for the cells in the calcein experiments shown in Fig. 4 [see Additional file 1].

For calcein influx measurements in regular and high-K^+^ Tyrode’s solutions, where z-stacks were available for each cell in the data set, the volume and concentration are determined more directly. First, the mean fluorescence for each cell slice at each z-step is normalized to the extracellular fluorescence, resulting in a mean calcein concentration for that cell at that z-step (*C*_*c*, *z*-*step*, *t*_).

3 $$ {C}_{c,\mathrm{z}-\mathrm{step},t}=\frac{F_{c,\mathrm{z}-\mathrm{step}}}{F_{extracellular,\mathrm{z}-\mathrm{step}}}\times 200\ \mu \mathrm{M} $$

Then, a summation of the mean calcein concentration multiplied by the cell area from all the z-steps gives the total number of molecules in the cell at each time point (*N*_*c*,*t*_).

4 $$ {N}_{c, pixelsum,t}={\sum}_{\mathrm{z}-\mathrm{step}}{C}_{c,\mathrm{calcein},\mathrm{z}-\mathrm{step},t}\ {A}_{c,\mathrm{pixel},t} $$

5 $$ {N}_{c,t}=\alpha\ {N}_{c, pixelsum,t} $$

Finally, the total number of intracellular calcein molecules is normalized to the initial volume of each cell, to get the change in concentration (*∆c*_*c*_) for a cell that does not change volume.

6 $$ {\Delta c}_c=\frac{N_{c,t}-{N}_{c,{t}_0}}{V_{c,{t}_0}}=\frac{N_{c, pixelsum,t}-{N}_{c, pixelsum,{t}_0}}{V_{c, pixelsum,{t}_0}} $$

### Normalization of changes in molecular concentration

For each of the three molecules, the change in intracellular concentration (*∆c*_*i*,*t*_) is calculated at each measurement time point by subtracting the intracellular concentration at time zero (*∆c*_*i*,0_) from the concentration at time *t*. The normalization of the concentration *∆c*_*i*, *t*_ is done with respect to the reference (*t* = 0) concentration for each measurement — that is, the initial extracellular dye concentration for influx measurements, and the initial intracellular dye concentration for efflux measurements.

### Membrane potential measurements

Cells were incubated with the membrane-potential-sensitive dye FluoVolt™ (Molecular Probes (Thermo Fisher Scientific)) at 1:500 dilution at room temperature for 30 min before washing and resuspension in RPMI 1640 for pulse exposure. For image analysis, a ring-shaped region enclosing the cell membrane was selected as the region of interest (ROI) for each cell. The change in membrane potential is proportional to the ratio of the change in mean fluorescence to the initial fluorescence (∆*F/F*_*0*_) in the ROI. Sham exposures were recorded with cells treated with the same protocol but without delivery of the pulsed electric field. Sham exposures were subtracted from each data point.

## Results

### Molecular transport of YO-PRO-1, propidium, and calcein

Figure 4 shows molecular transport, normalized in each case against the initial concentration difference, for YO-PRO-1, propidium, and calcein influx, and calcein efflux, following membrane permeabilization with 10, 6 ns, 20 MV/m pulses delivered at 1 kHz. We chose this electric field exposure to allow a measurable calcein influx. A comparison of the ratios of intracellular concentration change to the initial concentration difference is plotted in Fig. 4. To assure greatest accuracy, cell volume changes resulting from pulse-induced osmotic imbalance are taken into account in the calculation of dye concentrations [see Additional file 1]. Under these conditions, influx for the two positively charged dyes (YO-PRO-1, propidium) is significantly greater than influx of the negatively charged dye (calcein). By a similar factor, calcein *efflux* is greater than calcein influx.

**Fig. 5:**
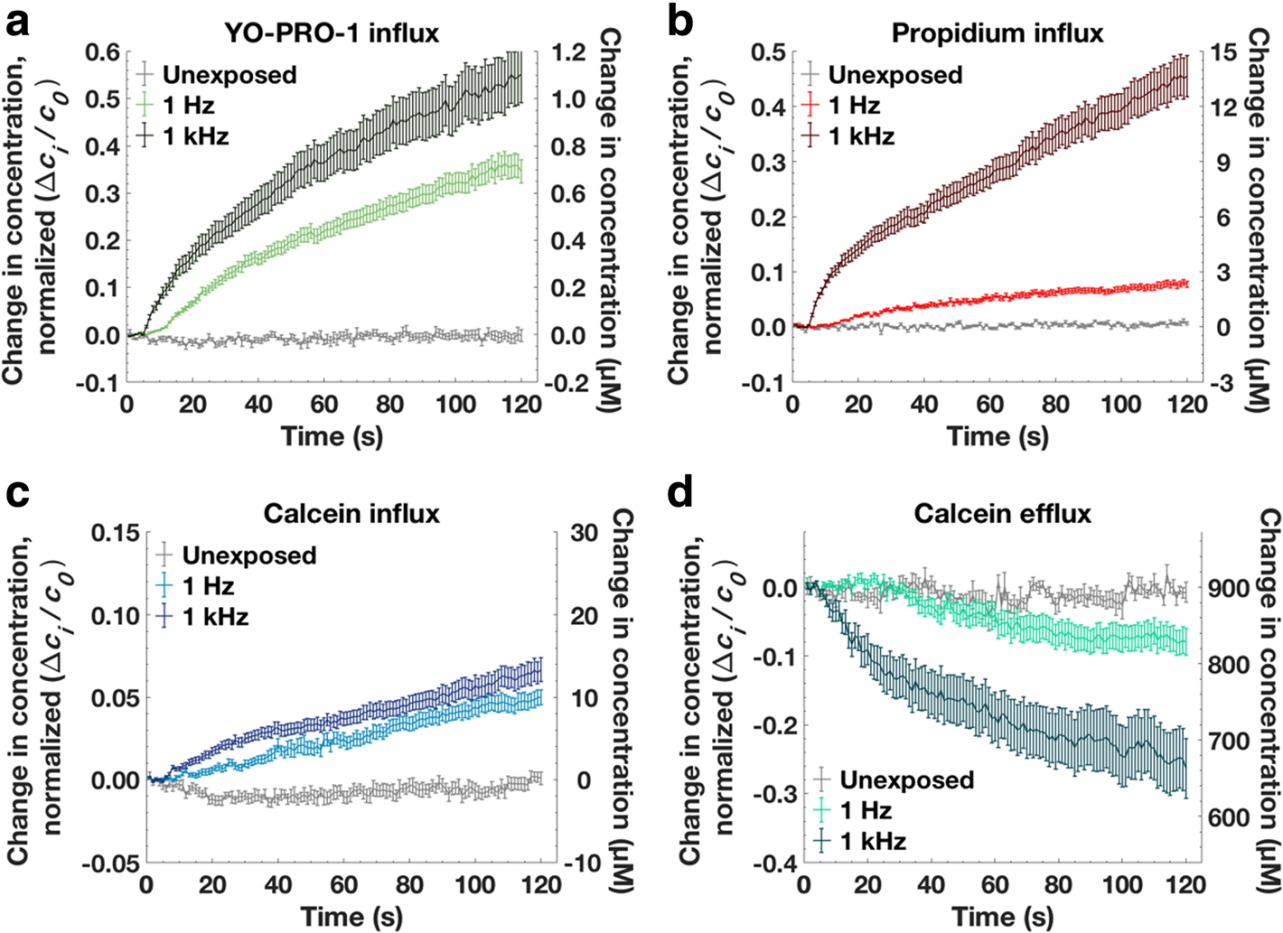
Measured molecular transport for U-937 cells after 10, 6 ns, 20 MV/m pulses at 1 Hz and 1 kHz repetition rates. Pulse delivery begins at t = 5 s. **a** YO-PRO-1 influx, (**b**) propidium influx, (**c**) calcein influx, (**d**) calcein efflux. Each curve represents three independent experiments with 20–34 cells measured for each condition

Figure 5 compares YO-PRO-1 influx, propidium influx, calcein influx, and calcein efflux after exposure to 10, 6 ns pulses delivered at 1 Hz and 1 kHz. (The 1 kHz data is the data plotted also in Fig. 4.) Concentration changes are given both normalized to the initial concentration difference (left y-axis) and in absolute concentration units (right y-axis).

Two minutes after pulse exposure the YO-PRO-1 intracellular concentration is about 1.1 μM for the 1 kHz case, and 0.75 μM for the 1 Hz case, corresponding to concentration change fractions of 0.55 and 0.38. ([YO-PRO-1]_extracellular_ = 2 μM.) Note the increase in the rate of YO-PRO-1 uptake after the tenth pulse delivered at 1 Hz (15 s into the recording). Similar increases in influx rate after the tenth pulse occur in the 1 Hz propidium and calcein data.

The intracellular propidium concentration reaches 13 μM after pulses delivered at 1 kHz, and 2.5 μM for 1 Hz pulses, corresponding to concentration change fractions of 0.43 and 0.08. ([propidium]_extracellular_ = 30 μM.)

Calcein influx is similar for 1 Hz and 1 kHz pulse repetition rates, with the intracellular calcein concentration after 2 min reaching 10–12 μM, a concentration change fraction of about 0.05. ([calcein]_extracellular_ = 200 μM.) Calcein efflux, however, is significantly greater for pulses delivered at 1 kHz (like propidium influx). The intracellular calcein concentration drops from 900 μM to 830 μM after the 1 Hz pulses, and to 660 μM after 1 kHz pulses, with corresponding normalized concentration change fractions 0.08 and 0.27.

### Membrane potential measurement with FluoVolt

Although current electroporation models assume that the transmembrane potential following a permeabilizing electric pulse exposure is effectively zero (cf. Introduction), the low level of calcein influx relative to YO-PRO-1 and propidium influx and to calcein *efflux* is consistent with a rapid, post-pulse reestablishment of the membrane resting potential. The recovering transmembrane potential, which is negative when the extracellular medium is taken as the reference potential, 0 V, favors the entry of cations into the cell but impedes the entry of anions (and facilitates their efflux).

**Fig. 6:**
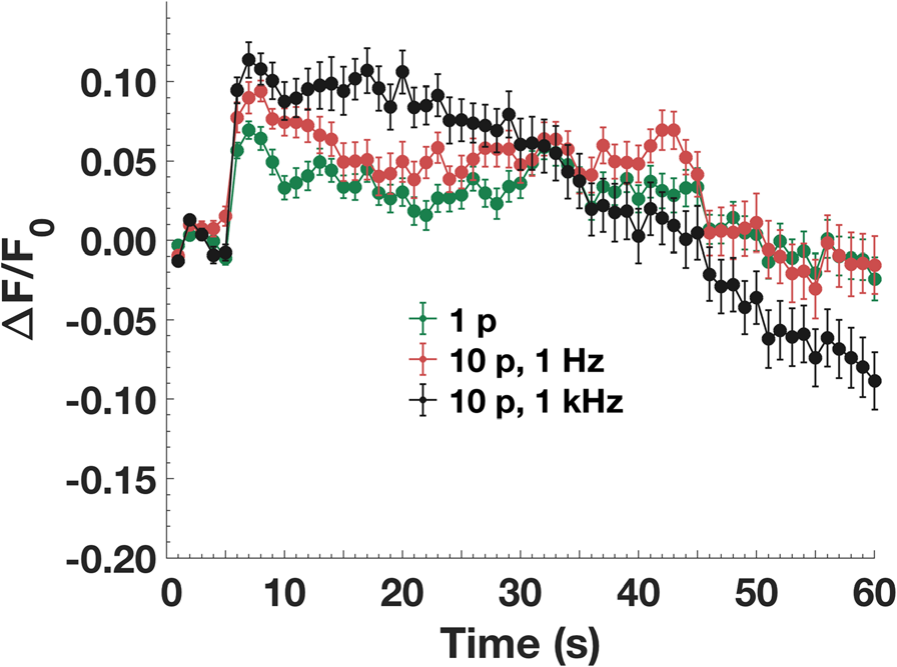
FluoVolt fluorescence change after exposure to a single pulse, or 10, 6 ns, 20 MV/m pulses at 1 Hz and 1 kHz repetition rates. Increased fluorescence indicates a decrease in the magnitude of the transmembrane potential. Pulse delivery begins at t = 5 s

To examine this hypothesis more directly, membrane potential measurements were carried out using the potential-sensitive fluorescent dye FluoVolt [39]. Figure 6 shows the measured fluorescence change after 1 and 10, 6 ns pulses at 1 Hz and 1 kHz repetition rates. Depolarization (fluorescence increase) is observed immediately after the pulse exposure in each case. 10 pulses (at 1 Hz) cause more depolarization than a single pulse. An initial partial recovery is observed 5 s after the exposure (10 s into the recording) in all cases. This partial recovery level is maintained for about twenty seconds, after which the fluorescence intensity returns to the pre-permeabilization level (less than 60 s after permeabilization). The FluoVolt fluorescence response after 10 pulses at 1 kHz — a much stronger permeabilizing dose than 10 pulses at 1 Hz (Fig. 5) — is difficult to interpret. It very likely indicates a more extreme disordering of the membrane, and of the FluoVolt molecules embedded in the membrane, which can no longer report in concert the composite transmembrane potential.

### Molecular transport in high K^+^ medium

To investigate further the contribution of membrane potential to post-pulse transport, calcein and YO-PRO-1 uptake experiments were carried out in physiological and high-potassium Tyrode’s solutions. Replacing sodium with potassium (145 mM K^+^) in the external medium impedes the cell’s ability to maintain its normal membrane resting potential [40]. High extracellular K^+^ also affects cell volume regulation [41], so it is essential to take volume changes into account when calculating the intracellular dye concentration. To reduce volume change and minimize blebbing and other morphological changes, we used a lower exposure dose (5, 6 ns, 20 MV/m pulses instead of 10), and we measured the cell volume using confocal z-stack measurements with calcein, as described in Methods. The average volume change after a 10-pulse exposure is 30%, compared to 5–10% after a 5-pulse exposure, as shown in supplemental Additional file 1: Figures S2 and S3, respectively.

**Fig. 7:**
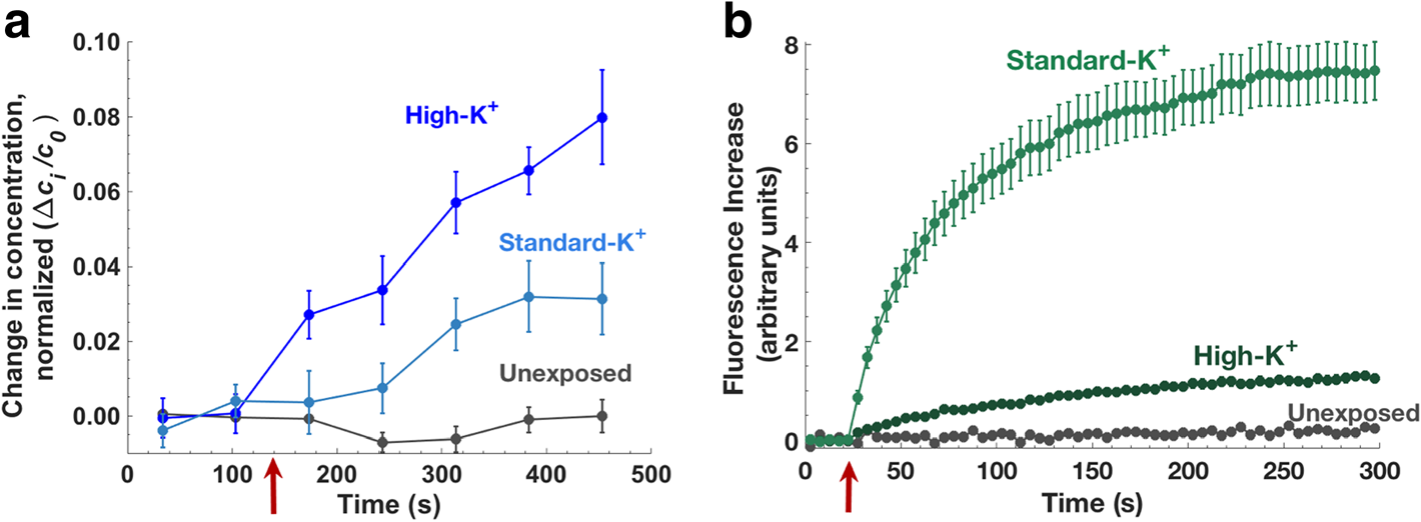
Influx in high and standard K^+^ Tyrode’s solutions after 5, 6 ns 20 MV/m pulses. (**a**) calcein (**b**) YO-PRO-1. Red arrows show the pulse delivery time

Figure 7 shows calcein and YO-PRO-1 influx with high and physiological [K^+^], corrected for changes in cell volume. Five minutes after pulse delivery, calcein uptake is 3–4 times higher in high-K^+^ Tyrode’s than in standard Tyrode’s, and YO-PRO-1 uptake is seven times lower, consistent with a role for transmembrane potential in driving the post-pulse transport of these charged species.

## Discussion

### Transport of charged small molecules across electropermeabilized membranes

Large differences between calcein efflux and influx have been reported previously [18, 42, 43], but without comment on the possible significance of the observation. To explain electroporation-induced cationic and anionic dye influx into cells, and influx and efflux of the anionic dye calcein, we propose that, contrary to the common assumption that the transmembrane potential is effectively zero after electroporation, the transport of charged substances across electropermeabilized membranes is driven by both concentration gradients and electric potential gradients. To our knowledge, although there are many descriptions of the kinetics of post-permeabilization membrane *conductance*, there are only two reports containing measurements of post-permeabilization membrane *potential*. One shows that the membrane resting potential in a patch-clamped cell can recover to at least 80% of its normal value within 90 s of the porating electric field exposure [14]. In the other, FluoVolt fluorescence indicates significant repolarization of the membrane within just a few seconds [28], consistent with the FluoVolt data presented here, and with one theoretical model [24].

Even a small non-zero membrane potential can have a large effect on the transport of charged molecules. To illustrate this, we calculate the pore-mediated transport of calcein using a simple electro-diffusive transport model. The Nernst-Planck equation combines two components of the current density, *J*_*s*_: diffusive (first term) and electrophoretic (drift, second term) [44]:

7 $$ {J}_s=-{D}_s\left(\frac{d{c}_s}{d x}+\frac{q_ez{c}_s}{kT}\ \frac{d\psi}{d x}\right) $$

where *D*_*s*_ is the diffusion coefficient, *c*_*s*_ is the local concentration, *q*_*e*_ is the elementary charge, *z* is the valence, and *ψ* is the membrane potential. Thus, we define *J*_*p,*_ electrodiffusive transport through a single cylindrical pore (without any interaction of the solute with the pore walls), as the sum of two corresponding components, the diffusion term (*J*_*diffusion*_) and the drift term (*J*_*drift*_):

8 $$ {J}_p={J}_{\mathrm{diffusion}}+{J}_{\mathrm{drift}} $$

9 $$ {J}_{\mathrm{diffusion}}=\frac{\uppi\ {r_{\mathrm{pore}}}^2{D}_s\ c}{l_{\mathrm{pore}}+\frac{\uppi\ {r}_{\mathrm{pore}}}{2}} $$

where *r*_*pore*_ is the pore radius, *l*_*pore*_ is the length of the pore (4.5 nm in these calculations), and *c* is the concentration difference from one side of the membrane to the other. And,

10 $$ {J}_{\mathrm{drift}}=\frac{1}{2}\frac{\uppi\ {r_{\mathrm{pore}}}^2{D}_s\ c}{l_{\mathrm{pore}}}\frac{q_{\mathrm{e}}z{V}_m}{kT} $$

where *V*_*m*_ is the transmembrane potential.

*J*_*s,p*_, electrodiffusive uptake accounting for solute-pore interactions can be described as [25, 32]:

11 $$ {J}_{s,p}\left[{\mathrm{pore}}^{-1}\ {\mathrm{s}}^{-1}\right]= HK{J}_p $$

where *H* and *K* are hindrance and partitioning factors respectively [32, 45] [Additional file 1].

The total solute transport for the entire cell membrane, *J*_*s,m*_, can be represented by including the exponential factor *N*_*pore*_(*t*), the time-dependent number of pores.

12 $$ {N}_{\mathrm{pore}}(t)={N}_{\mathrm{pore},0}{e}^{-t/\tau } $$

13 $$ {J}_{s,m}(t)={J}_{s,p}\ {N}_{\mathrm{pore}}(t) $$

For illustrative purposes, we choose an initial population of 6000 pores with a radius of 1.2 nm. The number of pores decays exponentially with a time constant *τ* = 50 s. These values, which fall within the wide boundaries predicted by current models, were chosen to produce a reasonable fit to our experimental data [see Additional file 1].

**Fig. 8:**
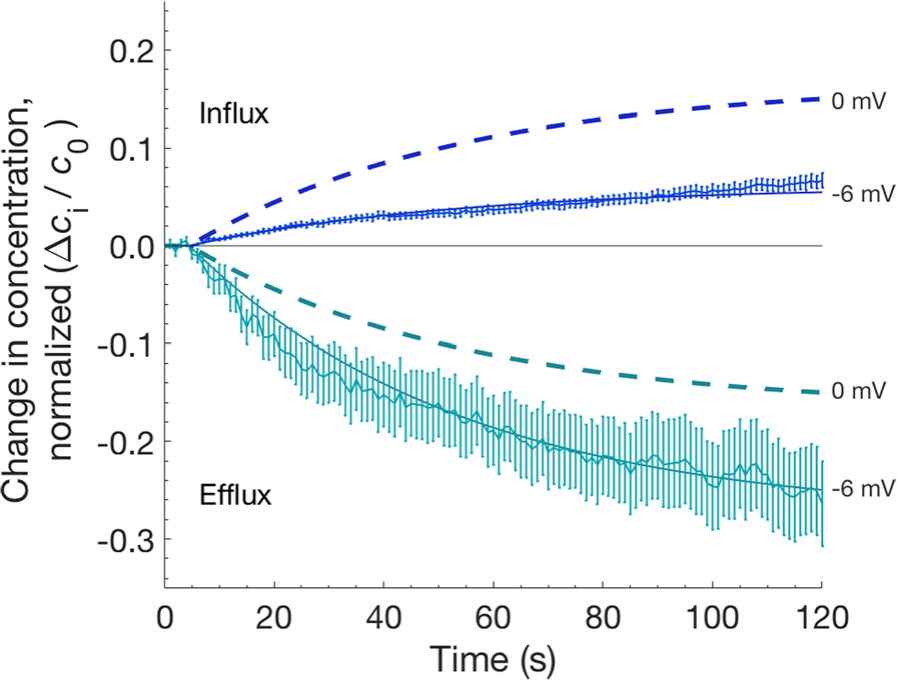
Measured and calculated electrodiffusive calcein transport. Solid lines are fit to the data shown in Fig. 4, using Eqs. 8–13, with *r*_*pore*_ = 1.2 nm, *N*_*pore,0*_ = 6000, *τ* = 50 s and *V*_*m*_ = − 6 mV. Dashed lines show the same calculations with *V*_*m*_ = 0 mV

Given these not unreasonable assumptions, a transmembrane potential of − 6 mV produces calcein transport (influx and efflux) comparable to our experimental observations (Fig. 8). Note that the set of permeabilizing structures in a real experimental population are very likely much more complex than a homogenous population of lipid pores with a single radius. For this example, however, it is sufficient to consider that the effective transport behavior is similar.

### Visualizing membrane depolarization and repolarization with FluoVolt

The immediate increase and subsequent rapid decrease in FluoVolt fluorescence observed after a permeabilizing pulse exposure is consistent with a pulse-induced depolarization of the membrane followed by repolarization within seconds. This interpretation, however, must be qualified. FluoVolt is an amphiphilic molecule with a hydrophilic fluorescent reporter that is connected to a hydrophobic electron-rich donor through a “molecular wire” [39]. To respond linearly and monotonically to the transmembrane potential, the FluoVolt molecule must be oriented with its long axis perpendicular to the plane of the membrane, and the fluorophore end of the molecule must be located at either the extracellular or intracellular face of the membrane, not both [39]. The disorganization of the membrane caused by a porating electric field very likely scrambles the orientation of the FluoVolt molecules along with other membrane constituents. For this reason, we cannot assume that FluoVolt reliably indicates membrane potential after an electropermeabilizing event.

### Modulating membrane potential and small molecule transport with high-K^+^ medium

YO-PRO-1 influx into electropermeabilized cells is greatly decreased in high-K^+^ medium, consistent with the hypothesis that transmembrane potential contributes to the post-permeabilization transport of charged small molecules by driving drift (electrophoretic) currents, both cationic and anionic.

Calcein influx, although greater in high-K^+^ medium, is affected less than that of YO-PRO-1. This may result from differences in molecular interactions of the two dyes with the membrane. A potentially useful index for these interactions is the molecular polar surface area (PSA). The PSA, which is used in drug discovery applications to predict molecular transport across membranes, is the sum of the surface areas of polar atoms in a molecule. In general, the higher the polar surface area, the lower the interaction with a lipid bilayer. Molecules with high polar surface area tend to be impermeant. Calcein’s higher polar surface area (230 Å^2^ versus 16 Å^2^ for YO-PRO-1) makes interactions with the membrane less favorable [46].

We have previously observed a significant dissimilarity in transport patterns for calcein and YO-PRO-1, and we have suggested that the electrostatic modifications in the membrane interface resulting from pulse-induced phosphatidylserine (PS) externalization [47–49] may in part be responsible for polarized transport patterns that were seen with YO-PRO-1 but not with calcein [50]. PS externalization may be partially blocked in high-K^+^(145 mM) buffer [51, 52], which would affect YO-PRO-1 transport more than calcein.

### Electroporation models and the electropermeome

Electroporation models that predict diffusive molecular transport based on evolution of a pore population that is governed by a pore energy landscape have provided mechanistic insights and guidance for investigations, and in response have evolved over the last three decades to accommodate experimental findings [25, 33, 53–56]. These models, to become not only descriptive but also predictive, must address, among other things, their inability to represent long permeabilization lifetimes in cells [14, 57], unreconciled estimates of pore sizes [20, 32, 36], and varying degrees and localization of permeability based on transport molecule identity [50, 58–60]. The evidence presented here indicates that electroporation transport models must also include the drift component of post-permeabilization electrodiffusion, which until now has been considered to be negligible. This could be done by adding a drift (electrophoretic) component to the model transport equations, and by incorporating parameters for the recovery of the transmembrane potential after the permeabilizing event.

It is very likely that the models will require further expansion, additional transport terms, and more parameters, especially for accurate representation of the long-lasting permeabilization observed with most electroporation protocols. Candidates for these persistent permeabilizing structures and processes include multidrug resistance protein channels and organic anion transporters, which are known to transport small molecules like calcein [61, 62], purinergic receptor channels [63], transient receptor potential (TRP) channels [64], pannexin channels [65], and other physiological components of a cell under stress.

This call for a substantial overhaul of existing electroporation models reflects a recognition of the need to accept and embrace the complexity of the system under analysis. The physical model of electric-field-induced defects in a dielectric shell is instructive and foundational, but it cannot begin to represent a living cell responding to the stress of abrupt osmotic imbalance, ion and metabolite leakage, membrane depolarization, and the loss of regulatory and structural compartmentalization. We call all the electropermeabilization-induced transport structures, and the resulting repair and restoration processes, and all the associated transmembrane traffic, the electropermeome. This is complexity that evolves, from the initial water defects and lipid pores observed in lipid bilayers, to the largely unexplored effects of porating electric fields on membrane proteins, to the stress- and damage-related activation of multiple signaling, repair, and reconstruction pathways that follow the loss of membrane integrity and the disruption of homeostasis [12].

## Conclusions

Post-electroporation influx of the cationic dyes YO-PRO-1 and propidium, and efflux of the anionic dye calcein, are all much greater than the influx of calcein after the same permeabilizing electric pulse exposure. These observations are consistent with a rapid recovery of the transmembrane potential in electropermeabilized cells, and a significant contribution from electrodiffusive transport. The kinetics of membrane depolarization and repolarization monitored with FluoVolt, and the effect of high-K^+^ medium on cationic and anionic small-molecule transport provide additional support for the hypothesis that post-electroporation transport includes a significant drift (electrophoretic) component, a feature not represented in current electroporation models.

## Additional file

Additional file 1: Cell volume change corrections and Electrodiffusive calculations: Details of image processing methods used for cell volume calculations and corrections; and detailed analytical calculations and parameters used to generate Fig. 8. (PDF 1192 kb)

## Abbreviations

DNA: Deoxyribonucleic acid
Pr: Propidium
PS: Phosphatidylserine
PSA: Polar surface area
RNA: Ribonucleic acid
ROI: Region of interest
TRP: Transient receptor potential
YP1: YO-PRO-1
