# Supplementary material for: Transport of charged small molecules after electropermeabilization — drift and diffusion
Source: BMC Biophys. 2018 Mar 21;11:4. doi: 10.1186/s13628-018-0044-2 (PMC5861730; doi:10.1186/s13628-018-0044-2)
Supplement: Supplementary file 1 — Cell volume change corrections and Electrodiffusive calculations: Details of image processing methods used for cell volume calculations and corrections; and detailed analytical calculations and parameters used to generate Fig. 8. (PDF 1192 kb) [file 13628_2018_44_MOESM1_ESM.pdf]

Transport of charged small molecules after electropermeabilization — Drift and diffusion

Esin B. Sözer[1], C. Florencia Pocetti[2], P. Thomas Vernier[1]

[1] Frank Reidy Research Center for Bioelectrics, Old Dominion University, Norfolk, VA 23508, USA

[2] Department of Bioengineering, Instituto Tecnológico de Buenos Aires, Buenos Aires, Argentina

Additional File 1

Cell volume change corrections

Measurements of cell volume changes were extracted from confocal z-stack images of cell suspensions containing 200 µM calcein in the extracellular medium (RPMI 1640) to enhance the definition of the cell perimeter. Calcein influx is very low, even after pulse exposure, so the fluorescence contrast between intracellular and extracellular regions is high. Stacks of 26, 1 µm z-slices were recorded every 70 seconds for over a period of 487 seconds, with pulse delivery at 140 s. (Each 26-slice z-stack takes 67 seconds). The mid-cell slice for each field of view was determined by focal adjustment for the largest cell cross-sections in the first fluorescence image in the recorded sequence. For the initial step in the image analysis, ROIs larger than the cell areas were determined manually in the transmitted light image using the 'imellipse' function of MATLAB (Figure 1a). These preliminary ROIs are intentionally larger than the cell cross-section for all time points. Precise cell boundaries within each ROI for each slice were then determined from the fluorescence images using the 'greythresh' function from the MATLAB image processing toolbox. 'greythresh' computes a global threshold intensity level that lies between 0 and 1 for images with regions of high contrast. Using this level in each cell ROI gives precise locations for the pixels comprising each intracellular area. These regions were then plotted and visually checked. Representative images from this procedure are shown in Figure S1.

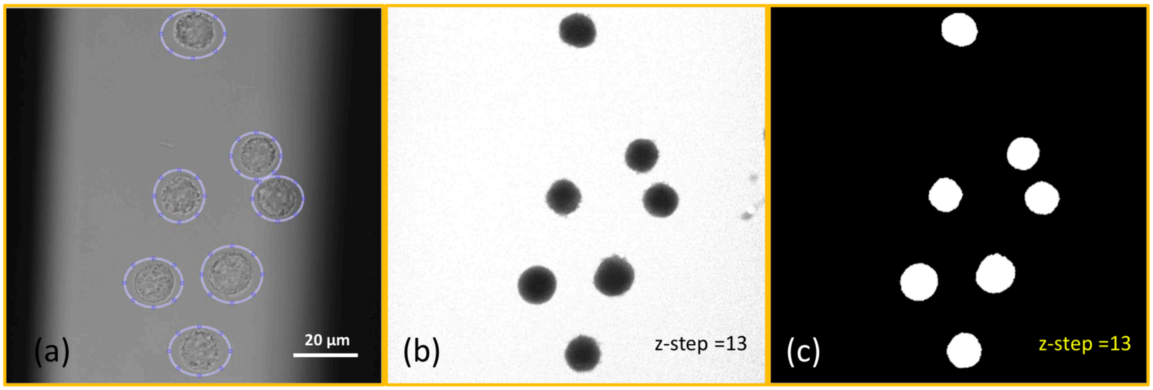

Figure S1 (a) Manually chosen ROIs that are intentionally larger than the cell boundaries shown on a representative transmitted light image. (b) Calcein fluorescence image of the mid-cell slice. (c) Cell regions determined with thresholding using MATLAB's greythresh function using fluorescent image (b).

The sum of the number of pixels for all slices for a given cell gives a number that is directly proportional to the volume of that cell. The ratio of this volume at any time to the volume at the beginning of the recording gives the volume change ratio (V_t/V_0).

For a perfectly spherical cell that swells or shrinks symmetrically in all directions, the volume change ratio is equal to the area change ratio in the mid-cell slice raised to the 1.5 power: $V_t / V_0 = (A_{t,mid\text{-}cell} / A_{0,mid\text{-}cell})^{1.5}$.

The actual relationship extracted from measurements of 26 U-937 (nonspherical, nonsymmetrical) cells from five experiments is $V_t / V_0 = (A_{t,mid\text{-}cell} / A_{0,mid\text{-}cell})^{1.2}$. We used this expression to convert area change ratio measurements to volume change ratios, in order to correct fluorescence intensities for volume changes, so that a volume change is not interpreted as a change in concentration of the indicator material. This procedure allows quantitative accounting for total material transport across the cell membrane.

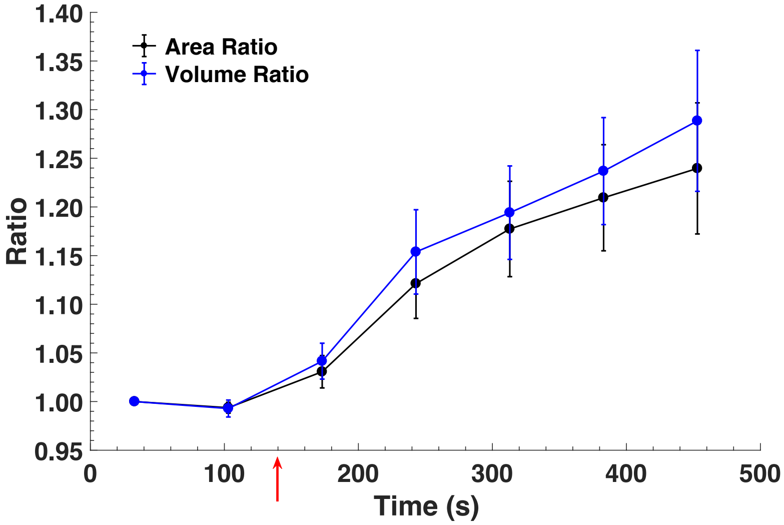

Figure S2 Change with time in area change ratio (A_t/A_0) and volume change ratio (V_t/V_0) after exposure to 10, 20 MV/m, 6 ns pulses, 1 kHz repetition rate (n = 26).

Similar measurements in high K^+ and normal K^+ Tyrode's buffer solutions were used for the analysis shown in Fig. 7 of the main text. The change in volume from these experiments is shown in Fig. S3 for both solutions.

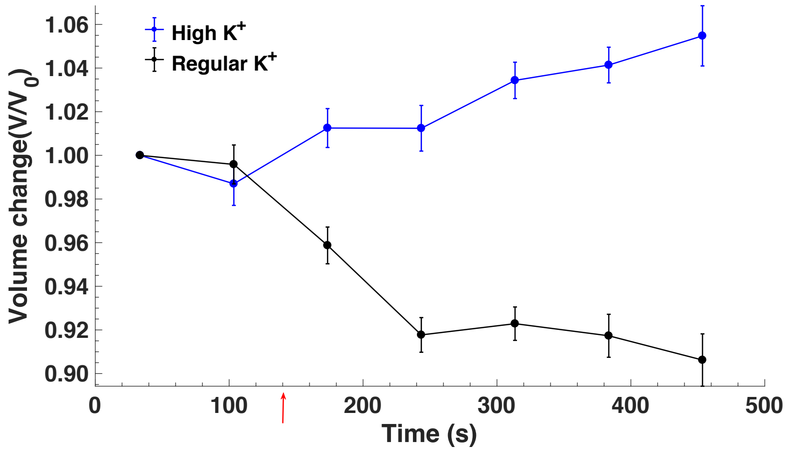

Figure S3 Volume change of cells exposed to 5, 6 ns, 20 MV/m pulses at 1 kHz in normal K^+ and high-K^+ Tyrode's solutions (n > 24).

Molecular transport before and after volume adjustment is shown in Figure S4.

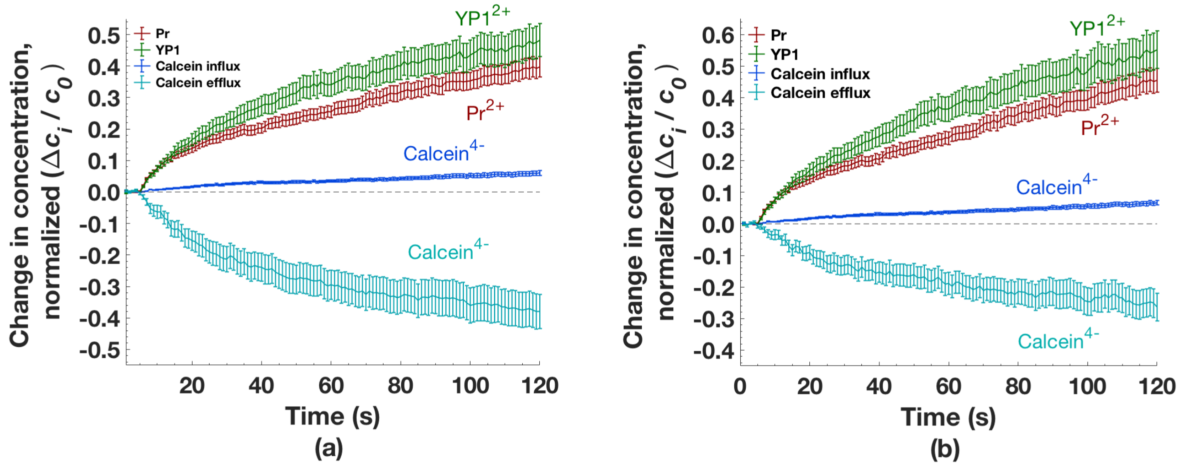

Figure S4 Normalized molecular transport of YO-PRO-1, propidium, and calcein (a) without correcting for pulse-induced cell volume change, (b) corrected for pulse-induced cell volume change.

Electrodiffusive calculations

This section provides more details for the calculations described in the Discussion subsection "*Transport of charged small molecules across electropermeabilized membranes*".

In the main manuscript, Equations 7-13 describe transport through pores of uniform size distribution, across the whole cell membrane. In Equation 11, we use hindrance and partitioning factors to calculate $J_{s,p}$, the electrodiffusive flux, accounting for pore-solute interactions **(Smith 2011, Smith and Weaver 2011, Son et al. 2014)**,

Hindrance (H) arises from two factors, decreased effective area when the solute is passing through the pore (f_A), and the drag exerted on the solute by the pore walls (f_D) **(Smith 2011)**. For a detailed explanation of the calculation of each factor, refer to Smith **(2011).** Here we give the specific equations used for the calculations that generated Fig. 8 in the main text.

$$H = \hat{f}_D f_A \tag{S6}$$

where effective area f_A is given by

$$f_A = (1-\lambda)^2 \tag{S7}$$

with $\lambda = r_s/r_{pore}$, where r_s is the radius of the smallest cylinder that can accommodate the solute volume, and $\hat{f}_D$ is the drag factor (f_D) modified for a cylindrical solute according to

$$\hat{f}_D = \frac{f_D}{f_D + (1-f_D)\frac{l_s}{2r_s}} \tag{S1}$$

where l_s is the height of the smallest cylinder that can accommodate the solute volume.

The drag term f_D is calculated as follows:

$$f_t(\lambda) = \frac{9}{4}\pi^2\sqrt{2}\,(1-\lambda)^{-5/2}\,(1 + a_1\,(1-\lambda) + a_2\,(1-\lambda)^2) + a_3 + a_4\lambda + a_5\lambda^2 + a_6\lambda^3 + a_7\lambda^4 \tag{S2}$$

$$f_D = \frac{6\pi}{f_t} \tag{S3}$$

with constants $a_1 = -1.2167$, $a_2 = 1.5336$, $a_3 = -22.5083$, $a_4 = -5.6117$, $a_5 = -0.3363$, $a_6 = -1.216$, $a_7 = 1.647$.

Partitioning (K) accounts for the energetic cost of moving a charged solute from a high dielectric constant medium to the low dielectric constant interior of a lipid bilayer.

$$K = \frac{\mathrm{A}}{B\,e^{-\gamma} - C} \tag{S4}$$

where A, B, C are factors given below and

$$\gamma = \frac{q_e\,z_s\,V_m}{kT} \tag{S5}$$

$$A = e^{-1+\gamma}$$

$$B = \frac{w_0\,e^{w_0 - n\gamma} - n\gamma}{w_0 - n\gamma}$$

$$C = \frac{w_0\,e^{w_0 - n\gamma} + n\gamma}{w_0 + n\gamma} \tag{S6}$$

with $n = 0.25$, and the Born energy

$$w_0 = \frac{5.3643\,(z_s\, q_e)^2}{kT} r_{pore}{}^{\alpha} \qquad \text{(S7)}$$

Equation S14 is an unpublished numerical derivation of Born energy computed for a cylindrical pore and used by **(Smith 2011)** with α = -1.803.

The total solute transport for the entire cell membrane, $J_{s,m}$, can be represented by including the exponential factor $N_{pore}(t)$, the time-dependent number of pores.

$$N_{\text{pore}}(t) = N_{\text{pore},0}e^{-t/\tau} \qquad \text{(S8)}$$

$$J_{s,m}(t) = J_{s,p}\, N_{\text{pore}}(t) \qquad \text{(S9)}$$

For the illustrative calculations of Figure 8, we assumed a uniform pore population with 1.2 nm size, which is reasonable based on MD simulations **(Levine and Vernier 2010)**. To extract a transport value that fits our measured data, we made small modifications to the exponential factor α in equation S14. The resulting hindrance and partitioning factors, together with other parameters used in these calculations, are listed in Table 1 for calcein and YO-PRO-1. Diffusion coefficients were calculated based on a geometric approximation as described in Sözer et al. 2017 **(Sozer et al. 2017, Supplementary Material)**.

Table 1 Parameters used for electrodiffusive calculations of Figure 8 (calcein) and Figure S4 (YO-PRO-1).

	Calcein	YO-PRO-1
r_s	0.58 nm	0. 53 nm
l_s	1.9 nm	1.71 nm
D_s	4.1×10^{-10} m²/s	4.5×10^{-10} m²/s
r_{pore}	1.2 nm	1.2 nm
$N_{pore,0}$	6000	6000
τ	50 s	50 s
H	0.03	0.05
α	-1.791	-1.838
$K(\alpha)$	0.03	0.05

In the electrodiffusive transport calculations for YO-PRO-1 shown in figure S5 below, a change in the transmembrane potential from V_m = 0 mV to V_m = -6 mV resulted in a 30% difference in total transport in two minutes.

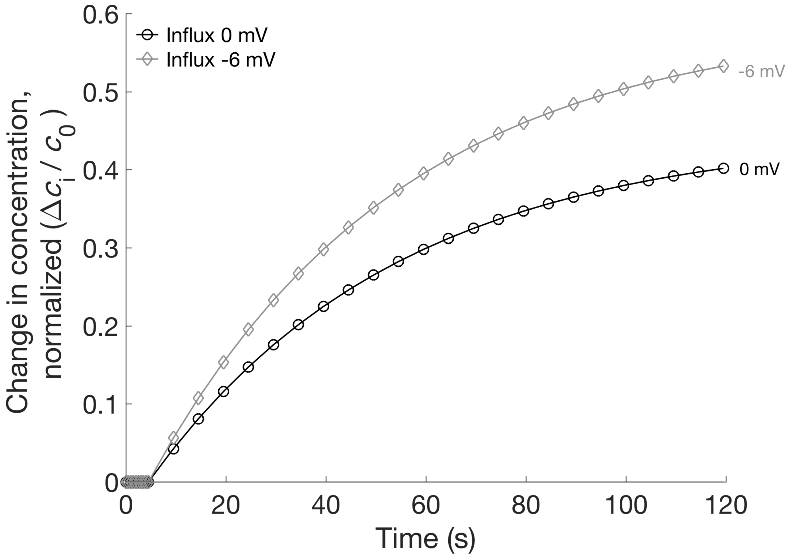

Figure S5 Calculated electrodiffusive calcein transport of YO-PRO-1 using equations 7-13 of the main manuscript and equations S1-9 with r_{pore} = 1.2 nm, $N_{pore,0}$ = 6000, τ = 50 s and V_m = -6 mV (diamond) or V_m = 0 mV (circle).

Levine ZA, Vernier PT (2010) Life cycle of an electropore: field-dependent and field-independent steps in pore creation and annihilation. J Membr Biol 236:27–36. doi: 10.1007/s00232-010-9277-y

Smith KC (2011) A Unified Model of Electroporation and Molecular Transport. Massachusetts Institute of Technology

Smith KC, Weaver JC (2011) Transmembrane molecular transport during versus after extremely large, nanosecond electric pulses. Biochem Biophys Res Commun 412:8–12. doi: http://dx.doi.org/10.1016/j.bbrc.2011.06.171

Son RS, Smith KC, Gowrishankar TR, Vernier PT, Weaver JC (2014) Basic features of a cell electroporation model: illustrative behavior for two very different pulses. J Membr Biol 247:1209–1228. doi: 10.1007/s00232-014-9699-z

Sözer EB, Levine ZA, Vernier PT (2017) Quantitative Limits on Small Molecule Transport via the Electropermeome — Measuring and Modeling Single Nanosecond Perturbations. Sci Rep 7:57. doi: 10.1038/s41598-017-00092-0
